# Improving Inhaler Technique through Face-to-Face and Multimedia Training: A QI Project in an Ethiopian Chest Clinic

**DOI:** 10.4314/ejhs.v35i1.6S

**Published:** 2025-12

**Authors:** Eman Omer Hassen, Hanan Yesuf, Dawit Kebede, Mahlet Fekadu Woldearegay, Shale Ali Kemal, Eyob Kebede Etissa, Eden Kahsaye Gidena, Yodit Abraham Yaynishet

**Affiliations:** 1 School of Medicine, College of Health Sciences, Addis Ababa University

**Keywords:** Asthma, Inhaler Technique, TASH, Ethiopia, Asthma Control, Dry Powder Inhaler, Metered Dose Inhaler, Pre- and Post-Intervention, Face-to-Face Demonstration, Multimedia

## Abstract

**Background:**

Although multiple studies have documented the prevalence of poor inhaler techniques in Ethiopia, no quality improvement initiatives have been conducted to provide guidance for improving inhaler techniques and symptom control. This quality improvement project aims to assess the effect of face-to-face demonstrations and multimedia-assisted demonstrations on improving inhaler techniques and symptom control among asthmatic patients attending the Chest Referral Clinic of Tikur Anbessa Specialized Hospital.

**Method:**

A total of 50 asthmatic participants using inhalers were recruited. Baseline asthma symptom control was assessed using the Asthma Control Test tool. Thirty of the recruited participants received face-to-face demonstrations from trained healthcare professionals while easy-to-use pictogram pamphlets and adapted videos were distributed. Inhaler techniques and asthma control were reassessed using adapted checklists and the Asthma Control Test, respectively, four weeks after the demonstrations and trainings were delivered. Finally, pre-intervention and post-intervention outcomes were analyzed and compared to quantify the impact of the implemented trainings.

**Results:**

A total of 30 participants underwent both pre- and post-intervention assessments of inhaler techniques and Asthma Control Test scores. There was a marked improvement in the proper use of inhalers, increasing from 10.5% to 52.6% among participants using dry powder inhalers (P = 0.007) and from 7.1% to 35.7% (P = 0.011) among those using metered dose inhalers.

**Conclusion:**

Face-to-face demonstrations of inhaler techniques, when supported with multimedia resources, significantly improved proper inhaler use among asthmatic patients. This project demonstrated a general trend toward better symptom control as measured by the Asthma Control Test post-intervention.

## Introduction

Inhalers are the most common method of administering asthma medication, with Metered Dose Inhalers (MDIs), Dry Powder Inhalers (DPIs), and Soft Mist Inhalers being the three major types. Improper use results in reduced medication effectiveness, affirming that incorrect inhaler device use is one of the major causes of uncontrolled asthma and frequent emergency department (ED) visits (1–3).

In a cross-sectional study conducted at TASH, 87.4% of patients demonstrated poor inhaler technique even though 79.3% of the participants reported having received instructions on how to use their inhalers (4). Furthermore, a small-scale cross-sectional study among pharmacy professionals in Addis Ababa revealed that 94% of professionals demonstrated poor technique (5).

This highlights a significant gap in understanding and operationalizing proper inhaler technique demonstration among healthcare providers.

Practice guidelines recommend that patient inhaler technique should be demonstrated and assessed at every clinical encounter using a checklist of critical steps, with repetition until competency is achieved (6). However, a substantial gap persists between guideline-recommended practice and real-world implementation.

Studies have shown that consistent education on inhaler technique reduces emergency room visits and asthma exacerbations (7). Person-to-person and multimedia-based trainings have been identified as the most effective approaches (8). Quality improvement projects dedicated to inhaler technique training were well received by patients, and following training, healthcare professionals were able to address patients' concerns more effectively. By the end of such projects, significant improvements in correct inhaler use and symptom control were observed (7–9).

A Cochrane systematic review of 29 parallel RCTs involving 2,210 asthmatic patients with follow-up periods ranging from 2 to 26 weeks demonstrated that enhanced inhaler technique education and multimedia training—including DVDs, computer applications, and games—improved technique in most studies both immediately after intervention and at follow-up, although the overall certainty of evidence was moderate due to risk of bias (8).

In this systematic review, three studies with 258 participants showed that 31 per 100 patients in the control group demonstrated correct technique compared with 69 per 100 in the education group (95% CI: 45 to 86). It also showed that 30 per 100 in the control group demonstrated correct technique compared with 47 per 100 in the multimedia group (95% CI: 26 to 70) (8).

A retrospective open cohort study conducted in a single hospital in Baltimore involving 525 patients with bronchial asthma and COPD (mean age: 71 years) assessed the effect of inhalation technique guidance and improvement procedures. Continuous repeated guidance steadily and significantly decreased errors with all inhaler devices (DPI, MDI, and soft mist inhalers) (P < .001 for all three device types). Elderly patients (>70 years) were found to particularly benefit from repeated guidance to reduce errors related to inhalation speed and gargling technique (10).

In a quality improvement project conducted in a hospital in New York City, 25 patients diagnosed with COPD and asthma were prospectively followed for 7 months. During each visit, they received education on inhaler technique. The intervention had a positive outcome in 84% of participants and no benefit in 16%, with a 91% reduction in ER visits among asthmatic patients after the intervention (7).

In a single-center QI project involving 30 COPD patients, it was demonstrated that a simple, rapid, and structured educational intervention—consisting of a teaching session complemented with a flyer—resulted in adequate overall inhaler use (80% of patients used their inhalers correctly), and this acquired competency remained stable for up to 14 days (83.0%, p > 0.99) (11).

This project aims to assess the effect of inhaler technique demonstrations (both multimedia and face-to-face) on improving MDI and DPI inhaler techniques and on symptom control using the ACT tool among asthmatic patients attending the Chest Referral Clinic of Tikur Anbessa Hospital from August to December 2023.

## Methods and Materials

**Sample size and design**: This is a quality improvement project in which assessments and data were collected before and after the intervention, and comparisons were made to quantify the impact of the intervention among the study population. As most literature and quality improvement projects that served as pilot studies worldwide included 25–35 patients, we enrolled 50 asthmatic patients with poor inhaler technique in this project, with a target sample size of 30 participants (7, 9, 12).

Although the target sample size was 30 participants, 50 patients were recruited to account for an anticipated 50% loss to follow-up due to the nationwide shortage of inhaler medications. Of these, 20 participants dropped out due to the national inhaler shortage, a viral upper respiratory tract infection epidemic during the study period, lack of transportation funds, and competing responsibilities. This may have introduced procedural bias, as only patients who maintained access to medications and could attend follow-up appointments remained in the study.

A limited number of patients were enrolled in this local quality improvement initiative, consistent with recommendations for rapid process improvement (23).

**Procedures**: The first eligible bronchial asthma patient presenting for follow-up at the TASH Chest Clinic at the beginning of the study period was recruited, and all consecutive patients were included until the sample size reached 50. Inhaler technique for each patient was assessed using a checklist (Annex I) adopted from previous studies by a trained general practitioner and the project lead in the OPD, along with completion of the Asthma Control Test (ACT) tool (Annex III) to assess baseline control for later comparison.

This was followed by face-to-face demonstrations of guideline-recommended MDI and DPI inhaler techniques delivered by the trained general practitioner and/or the primary investigator. The face-to-face demonstrations were delivered in groups of 5–15 participants per session. Training included the “teach-back method,” a communication strategy in which the healthcare professional demonstrates the technique and asks the patient to explain and repeat the procedure in their own words. Participants practiced until achieving 100% competency and asked questions. These demonstrations were given the waiting area of the bronchoscopy room of TASH. Each session lasted 1–3 hours depending on the number of participants. The demonstrations were supported by a pictogram pamphlet adapted from a previous study in South Africa and translated into Amharic (Annex IV). Designed for populations with low literacy and older age groups, the pamphlet was explained and distributed to all participants. Online videos that were adapted and translated into Amharic were distributed to participants with access to smartphones and social media. Patients were evaluated again 4 weeks after the demonstrations. Inhaler technique was reassessed at follow-up, with corrective feedback provided. The ACT questionnaire was also completed again. Questionnaires adopted from previous studies evaluating the convenience and feasibility of videos and pamphlets were completed 4 weeks after the intervention (Annex III).

**Design–to–action plan**: The project followed the Institute for Healthcare Improvement's Plan–Do–Study–Act (PDSA) cycle.

- **Plan**: Fishbone analysis and literature review were done to identify evidence-based interventions on improving MDI and DPI technique. The most practical interventions were face-to-face demonstrations and promoting documentation of inhaler technique at each visit in accordance with GINA guidelines.
- **Do**: Training sessions and demonstrations followed by reassessment of inhaler technique and asthma symptom control 4 weeks later.
- **Study**: Measurement and analysis of the impact of the intervention on asthma symptom control and inhaler technique, and identification of barriers to optimal education.
- **Act**: Implement periodic inhaler technique demonstrations and trainings for patients and healthcare providers, and incorporate structured documentation of inhaler technique for all asthma patients during each OPD visit using guideline-recommended checklists in the electronic medical record.

**Data collection**: Baseline characteristics and comorbidities were collected using a structured questionnaire (Annex II) from previous studies. Written informed consent was obtained before data collection. Inhaler technique was recorded before and after the intervention using a checklist (Annex I) adopted from previous studies. Patients missing any “essential” steps were categorized as having poor inhaler technique.

The Asthma Control Test Tool (Annex III) was also completed before and after the intervention to assess asthma control based on GINA guidelines. The ACT consists of four symptom/reliever questions plus a patient-rated control score. The minimum clinically important difference is 3 points. The use of the ACT for evaluating asthma control is supported by previous studies (13).

Videos and pictogram pamphlets adapted from previous studies (Annex IV) were distributed, and a feasibility and convenience questionnaire was completed afterward.

### Operational definitions

- **Asthma**: Diagnosed by pulmonologist and/or confirmed by spirometry.
- **Inhaler technique**: Steps used to deliver inhaled medications effectively to the lungs.
  – **Proper inhaler technique**: Completion of all essential steps (Annex I)
  – **Poor inhaler technique**: Omission of any essential step
  – **Essential steps**: Steps without which the drug cannot adequately reach the airways and efficacy is significantly reduced
- **Asthma control test tool**: A questionnaire assessing asthma symptom control over the past month; total score ranges 5–25 (Annex III)
  – Well-controlled: 20–25
  – Not-well controlled: 16–19
  – Poorly controlled: 5–15
- **Monthly income**: Categorized according to the 2020 WHO report:
  – Low (<3,100 birr)
  – Low-moderate (3,100–12,100 birr)
  – Moderate (12,101–37,600 birr)
  – High (>37,600 birr)
- **Trained patients**: Patients who received face-to-face demonstration at the Bronchoscopy Room of Tikur Anbessa by the project lead, residents, or other GPs specifically for asthma education.
- **Patients with multimedia assistance**: Patients who used either the pamphlet or instructional videos during inhaler use to aid in technique.
- **Teach-back method**: A communication strategy in which a healthcare professional explains and demonstrates a technique and asks the patient to repeat and perform the steps in their own words.

**Data analysis**: The collected data were checked, compiled, labeled, and cleaned before being entered into EPI software. Statistical analysis was performed using SPSS version 26. Descriptive analysis was conducted using mean, median, frequency, and percentage. After testing assumptions and distribution normality using the Shapiro–Wilk test, pre- and post-intervention inhaler technique total scores and ACT scores were compared using the Wilcoxon rank test.

A non-parametric test was selected because inhaler technique data were not normally distributed (Shapiro–Wilk: pre-intervention p = 0.049; post-intervention p = 0.001), and histogram visualization confirmed a negatively skewed distribution. Therefore, data were summarized using median and interquartile range (IQR), as mean and standard deviation are susceptible to influence by extreme values.

For the ACT score, normality was confirmed (Shapiro–Wilk: pre-education p = 0.570; post-education p = 0.396), and a paired sample t-test was used.

The influence of age, gender, education, and sociodemographic characteristics on improvement in inhaler technique was assessed using non-parametric tests. Categorical variables were compared with continuous variables using the Mann–Whitney test or the Kruskal–Wallis test, depending on subgroup number. Results are presented in tables and graphs. A p-value less than 0.05 was considered statistically significant.

**Ethical considerations**: Ethical approval was obtained from the Research and Ethics Committee of the Department of Internal Medicine, College of Health Sciences, Addis Ababa University. All patients provided informed consent (Annex V) to participate in the confidential interview. All patients also provided informed consent to participate in the intervention and follow-up assessment. All study procedures adhered to the ethical standards of the 1975 Helsinki Declaration, revised in 1983. Confidentiality and patient safety were ensured by excluding personal identifiers from all documents. Data were stored securely to prevent unauthorized access. All participants—regardless of performance during teach-back sessions—received compensation to partially cover time and transportation costs.

## Results

**Socio-demographic characteristics of participants**: Out of the 50 asthmatic participants who were recruited and evaluated at the beginning of the project, 20 participants were excluded. A total of 30 participants were included in the final analysis. The high attrition rate was attributed to the nationwide shortage of inhalers, lack of transportation, and upper respiratory tract epidemics.

**Asthma-related characteristics of the study participants**: Of the total study participants, 11 (36.7%) were MDI users, 2 (6.7%) were DPI users, and 17 (56.6%) used both inhalers.

**Characteristics of pre-education inhaler technique**: Twenty-eight participants were evaluated for their MDI technique, and only 7.1% demonstrated proper technique. Among the 19 participants who were evaluated for their DPI technique, only 10.5% demonstrated proper inhaler technique. In the DPI-using group, Step 1 (“Remove the cap from the inhaler”) and Step 4 (“Rotate grip until click is heard”) were correctly completed by all patients (n = 19). Step 5 (“Exhale away from the mouthpiece”) was the most frequently missed step. Additionally, the step “Exhale to residual volume and hold breath for 5 seconds” was correctly practiced by only 21.1% of participants.

In the PMDI-using group, the most correctly performed step was Step 1: “Remove mouthpiece cover and shake” (28, 100%), while the most missed step was Step 3: “Exhale to residual volume” (5, 17.2%).

**Improvement of asthmatic participants' inhaler technique after training**: In this study, 63.3% (n = 19) of the participants used MDI technique. Accordingly, essential steps such as “Keeping the inhaler upright” improved from 94.7% to 100%, “Exhaling to residual volume” improved from 21.1% to 78.9%, and “Exhaling away from the mouthpiece” improved from 5.3% to 100%. The overall proper MDI inhaler technique increased from 5.3% to 52.6%.

Using the non-parametric Wilcoxon Signed Rank test, the median score of the total number of inhaler technique steps increased from a baseline of 6 (IQR 5, 6) to a median of 8 (IQR 7, 9), with a Z-score of −4.23 (P < 0.001) one month after the intervention involving face-to-face education augmented with a pictogram pamphlet. Five out of nine MDI steps showed significant improvement, while the remaining four were mostly correct at baseline.

On the other hand, 93.3% (n = 28) of participants used MDI inhaler technique in both pre- and post-multimedia practice. Essential steps such as “Holding the inhaler upright” improved from 82.1% to 100%, and “Exhaling to residual volume” improved from 17.9% to 64.7%. The overall proper PMDI technique increased from 7.1% to 35.7%.

DPI technique also showed a statistically significant improvement, with the baseline median of 6 (IQR 4, 7) increasing to 8 (IQR 8, 9), Z-score = -3.75, P < 0.001. Six out of nine DPI steps demonstrated significant improvement, while the remaining three were mostly correct at baseline. The number of participants demonstrating proper inhaler technique without missing any essential steps increased from 2 (10.5%) to 10 (52.6%) for DPI users (P = 0.007), and from 2 (7.1%) to 10 (35.7%) for MDI users (P = 0.011).

**Characteristics of pre- and post-education Asthma Control Test scores**: According to the ACT tool, pre-intervention 56.7% of participants had a score of <15, 33.3% scored between 16–20, and only 10% had a score of >20.

Findings showed that the proportion of patients with poorly controlled asthma decreased from 56.7% to 46.7% after face-to-face demonstrations aided with pictograms, while the well-controlled group increased from 10% to 23.3%. The pre-intervention median ACT score of 13.5 (IQR 10.75, 18) increased to 15 (IQR 11, 18.5) post-intervention. However, this increase in ACT score was not statistically significant (P = 0.074).

**Multimedia use among participants**: Among the participants, 63.3% used pamphlet pictograms, 23.3% used both pamphlet and video demonstrations, and 13.3% used neither. Among those who did not use video demonstrations, 100% reported not owning a smartphone. This barrier could not be addressed due to lack of financial resources to provide smartphones.

Among those who did not use pamphlet pictograms, 43% reported losing the pamphlet and 57% reported being unable to read and understand it. To address this barrier, more time was allocated to explain each picture and its context for participants who had difficulty understanding it. Among participants who used the pamphlet pictograms, 83.3% reported that the contents were easy to understand, and 80% believed that the pamphlets helped them improve their inhaler technique.

## Discussion

From this project, we demonstrated significant improvement in proper inhaler technique (P < 0.001) for both MDI and DPI users after receiving face-to-face demonstrations supported with pictogram pamphlets.

This is the first reported pilot quality improvement project in Ethiopia in which face-to-face trainings assisted with multimedia demonstrations led to measurable improvement in both MDI and DPI inhaler techniques.

The median MDI technique score and overall proper MDI inhalation technique increased post-intervention. This finding aligns with a pilot quality improvement study conducted in South Africa, which reported an increase in mean MDI technique score from 4.6 ± 2.2 to 7.9 ± 2.7 (P < 0.05) after implementing a study leaflet augmented with MDI demonstrations.

Use of DPI techniques also improved significantly, with 52.6% of DPI users demonstrating correct technique at four weeks post-intervention. A similar improvement was reported by Marando et al., where 80% of patients demonstrated correct DPI technique, with results sustained for two weeks after structured leaflet-based demonstrations.

Participants were five times more likely to demonstrate proper technique for both DPI and MDI inhalers after receiving face-to-face demonstrations augmented with pamphlets. This confirms the effectiveness of face-to-face demonstrations in group settings using a teach-back method supported with multimedia—particularly pictogram pamphlets—on improving inhaler technique in asthmatic patients.

This educational intervention showed clear success by producing statistically significant improvements in several MDI and DPI technique steps and by increasing the total number of correctly executed steps post-intervention. Although ACT scores did not show statistically significant improvement post-intervention, numerical results indicated a decrease in poorly controlled asthma and a general trend toward better symptom control. Basheti et al. reported statistically significant ACT score improvement (P = 0.003) after educational intervention, with increased complete asthma control at 26 weeks of follow-up. Similar findings were reported in a Spanish study that followed 25 patients over seven months after educational intervention.

Although small sample size and short follow-up duration may have contributed to the lack of statistically significant improvement in ACT score in this project, the observed trend favors improvement in symptom control.

Age, education level, and previous inhaler technique instruction were not significantly associated with improved inhaler technique, in contrast to previous studies. This discrepancy can be explained by the small sample size, which limits the ability to detect such associations.

The small sample size and short four-week follow-up period limit conclusions regarding long-term retention. Future studies with longer follow-up are recommended to assess durability of learning. Nonetheless, similar studies have been conducted with comparable sample sizes and even shorter follow-up periods of only two weeks. The limited number of participants in this quality improvement project is also consistent with recommendations for rapid process improvement.

The project was conducted in a single center, and selection bias may have occurred because only asthmatic patients attending the Chest Clinic of TASH were included. The use of multiple interventions makes it difficult to determine which one contributed most to the observed improvement because subgroup analysis was limited by the small sample size. In addition, the nationwide shortage of inhaler medications discouraged participation and contributed to the high dropout rate.

Despite these limitations, this is, to our knowledge, the first reported quality improvement project in Ethiopia that demonstrated a significant increase in proper MDI and DPI inhaler technique. The project highlights that face-to-face group demonstrations using a teach-back method, complemented by pictogram pamphlets, can produce adequate inhaler proficiency and maintain it over a four-week follow-up period.

We recommend using this pilot project as a basis to implement regular inhaler-technique training for asthmatic patients. Trained health professionals should provide face-to-face group demonstrations using a teach-back method at regular intervals, and pictogram pamphlets should be used to reinforce learning.

Furthermore, assessment of inhaler technique using a checklist of critical steps should be performed at every patient encounter, and documentation of inhaler technique should be completed at each visit. We strongly recommend that this pilot project paves the way for extending similar quality-improvement initiatives to other hospitals serving asthmatic patients and ultimately contributes to the development of nationwide strategies for improving inhaler technique among all patients using inhalers in Ethiopia.

## Figures and Tables

**Table 1:** The socio-demographic characteristics of the study participants among asthmatic patients in the Chest Referral Clinic of Tikur Anbessa Specialized Hospital, 2023

Characteristics	Frequency	Percent
Age in years		
18-30	1	3.3
31-50	12	40
51-70	16	53.4
>70	1	3.3
Sex		
Male	11	36.7
Female	19	63.3
District residence		
Urban	29	96.7
rural	1	3.3
Marital status		
Single	8	26.7
Married	18	60
Divorced	3	10
widowed	1	3.3
Education level		
no formal education	5	16.7
primary	10	33.3
secondary	7	23.3
higher education	8	26.7
Occupation		
Government employee	9	30.0
private employee	9	30.0
self-employee	1	3.3
Unemployed	6	20.0
Retired	3	10.0
Housewife	2	6.7
monthly income		
Low (<3100birr	12	40.0
low-moderate (3100-12100)	17	56.7
moderate (12101-37600)	1	3.3

**Figure 1:**
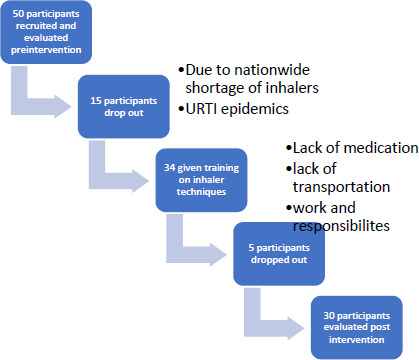
Study Participant enrollment

**Figure 2:**
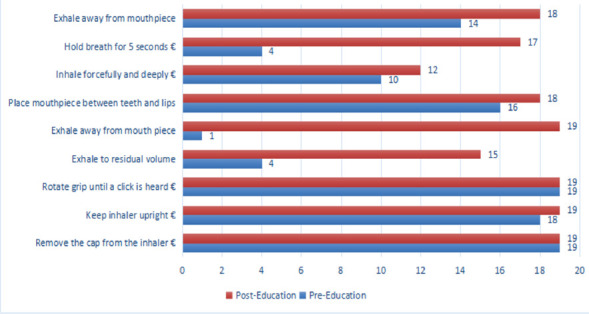
DPI technique compared among participants pre- and post-intervention as compared by the color coding specified above Pre-intervention Median Score: 6 (IQR 4,7) Post-intervention Median Score: 8(IQR 8,9 Improvement-Zscore = -3.7; P<0.000; N= 19

**Figure 3:**
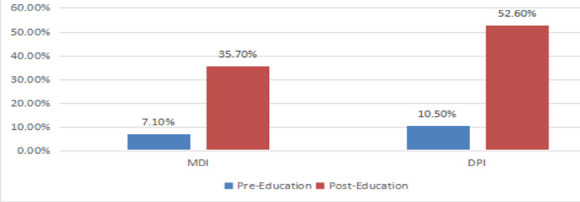
Percentage depiction of proper inhaler technique in DPI and MDI users pre- and post-education as color coded above MDI N= 28; DPI N= 19

**Figure 4:**
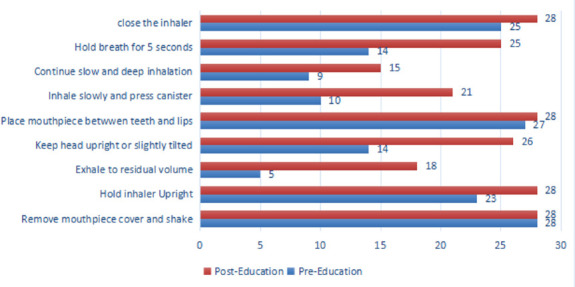
MDI technique compared among pre- and post-intervention as color coded above Pre-intervention Median Score: 6 (IQR 5.6; Postintervention Median Score: 8(IQR 7,9); Improvement: Z-score=-4.25, P-value-<0.0001; N= 28

**Figure 5:**
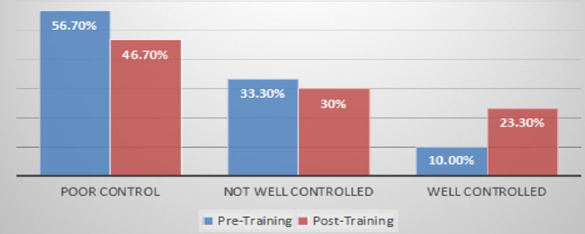
ACT tool score graded pre- and post-education Sample size= 30 participants
